# The Predictive Role of Video Head Impulse Testing Patterns of Anti-Compensatory Saccades Using the Suppression Head Impulse Paradigm for the Diagnosis of Mild Acute Unilateral Vestibular Loss

**DOI:** 10.3390/audiolres15050110

**Published:** 2025-08-30

**Authors:** Cristiano Balzanelli, Fabio Pontara, Luca Oscar Redaelli de Zinis

**Affiliations:** 1 Vertigo Center, San Bernardino Outpatient Clinic, 25087 Salò, Italy; 2 Department of Otorhinolaryngology and Audiology, University of Ferrara, 44121 Ferrara, Italy; 3 Department of Medical and Surgical Specialties, Radiological Sciences, and Public Health, Section of Audiology and Phoniatrics, University of Brescia, 25123 Brescia, Italy

**Keywords:** VHIT, SHIMP, anti-compensatory saccades, AUVL

## Abstract

**Background/Objectives:** To evaluate the predictive role of anti-compensatory saccades in diagnosing mild acute unilateral vestibular loss. **Methods:** Consecutive patients with vertigo who underwent a bedside vestibular evaluation and video head impulse testing of the horizontal semicircular canal at the San Bernardino Outpatient Clinic in Salò, Italy, between 1 January and 30 June 2024 were examined (Group 1). Two control groups were considered: patients with severe unilateral acute vestibular loss (Group 2) and healthy subjects (Group 3). The video head impulse testing patterns of anti-compensatory saccades (amplitude, scattered pattern, and latency) using the suppression head impulse paradigm were analyzed to evaluate their predictive role in identifying horizontal canal dysfunction, even when borderline gain values of the canal’s vestibulo-ocular reflex were present. **Results:** Group 1 included 74 patients, Group 2 included 20 patients, and Group 3 included 20 healthy, voluntary subjects. The anti-compensatory saccades revealed significant differences in the amplitudes and scattered patterns between the two ears, exclusively in Groups 1 and 2. **Conclusions:** The anti-compensatory saccades alterations using the suppression head impulse paradigm can predict mild acute unilateral vestibular loss, even when the horizontal semicircular canal’s vestibulo-ocular reflex gain values are mild (lower borderline). Conversely, a borderline asymmetry of the horizontal semicircular canal’s vestibulo-ocular reflex using the classic head impulse paradigm should not be considered a marker of mild acute unilateral vestibular loss when the saccadic pattern is symmetrical using the suppression head impulse paradigm. Further meticulous differential diagnostic investigations are necessary in such cases to effectively diagnose horizontal semicircular canal dysfunction.

## 1. Introduction

Acute unilateral vestibular loss (AUVL) is considered one of the most common causes of vertigo [1,2]. It is estimated to be the second most common cause after benign paroxysmal positional vertigo (BPPV), with an incidence rate ranging from 3.5 to 20.2 per 100,000 people [3,4]. AUVL accounts for 3 to 10% of all specialist vertigo diagnoses [5].

Since the introduction of video head impulse testing (vHIT) [6], it has been possible to quickly and accurately assess the vestibular function at high frequencies (HFs) of head impulsive stimulation (5–6 Hz) in all three canals of each labyrinth. However, despite this significant technological advancement, cases of diagnostic uncertainty persist, primarily due to the lack of symptomatic, clinical, or instrumental findings. The development of the “Suppression Head Impulse Paradigm” (SHIMP) has increased the reliability of diagnosing horizontal semicircular canal (HSC) dysfunction with the conventional “Head Impulse Paradigm” (HIMP) [7,8,9]. In this stimulation modality, the target (laser point) is “head-fixed” rather than “earth-fixed”, and it moves consensually with the head, generating a wide anti-compensatory saccade (AS) as the head abruptly stops, with a delay of about 80 milliseconds (ms). An AS is defined as an eye’s movement in the same direction as the head’s movement, aiming to recover the target [8].

In AUVL, the amplitude of the AS is directly proportional to the gain values up to the point of complete disappearance in the most severe cases. According to some authors, AS alterations appear to be directly proportional to the VOR gain deficits, subjective vertigo intensity, low VOR gain values, and the amplitude or gathered/scattered pattern of compensatory saccades (CSs) obtained using the HIMP-vHIT [10,11,12]. Some alterations in AS patterns are directly proportional to the degree of partial or chronic uncompensated vestibular loss, thus serving as an effective diagnostic indicator in the acute phases of both severe and even mild vestibular dysfunction.

This study aims to evaluate the effectiveness of using SHIMP to detect borderline HIMP-vHIT diagnoses or mild AUVL (mAUVL) by analyzing AS pattern variations.

## 2. Materials and Methods

This retrospective study examined patients with mild vertigo symptoms evaluated at the Vertigo Center of the San Bernardino Outpatient Clinic in Salò, Italy, between January and June of 2024 (Group 1). The control groups consisted of patients with severe AUVL (sAUVL) (Group 2) and healthy subjects who had never previously experienced vertigo (HSs) (Group 3). All the patients underwent a comprehensive medical history review, complete bedside vestibular testing, infrared video-oculoscopy, and vHIT for the horizontal canal, both in the HIMP and SHIMP modes.

The Group 1 inclusion criteria for mAUVL were documented in the patients’ recent histories and physical examination findings.

SYMPTOMS:

- Dizziness (not vertigo).
- Nausea (not vomiting).
- Unsteadiness and non-rotational vertigo (not a fall).

BEDSIDE TESTING:

- Slight unidirectional rotation on the Unterberger test (less than 30 degrees of rotation in 50 steps).
- Positive past-pointing test (unilateral deviation on the Barany outstretched arms test).
- No evidence of spontaneous nystagmus (infrared video goggles evaluation).
- Slight rhythmic, conjugated, persistent (not paroxysmal), horizontal, unidirectional nystagmus on positioning testing (infrared video goggles evaluation).
- Nystagmus inhibited by fixation (infrared video goggles evaluation).
- Positive HIT (head impulse test) outcomes were considered doubtful.
- Negative HST (infrared video goggles evaluation).

VHIT TESTING:

- Borderline lower VOR gain values for the affected side (as is known, normal VOR gain values for the horizontal canal are 0.8–1.0; we considered “lower borderline VOR gain values” to be 10% less than 0.8, which means a range of 0.72–0.8).
- Borderline asymmetry of the VOR gain values on the vHIT in HIMP mode was observed (we considered all cases with less than a 20% discrepancy between the two sides, even when the VOR gain was within the normal range of 0.8–1.0).

The Group 1 exclusion criteria for mAUVL were as follows:

SYMPTOMS:

- Long-lasting vomiting.
- Rotational vertigo.
- Falls, ataxia, and dysmetria.

BEDSIDE TESTING:

- Unterberger test: Rotation of more than 30 degrees in 50 steps.
- Spontaneous first-, second-, and third-degree nystagmus in the sitting position.
- Clearly positive HIT.
- HST positivity (infrared video goggles evaluation).
- Simultaneous diagnosis of BPPV (infrared video goggles evaluation).
- Paroxysmal, arrhythmic, deconjugated, or non-inhibited by fixation nystagmus (infrared video goggles evaluation).
- Other signs and/or symptoms of pathologies of central, vascular, metabolic, traumatic, iatrogenic, or malformative origin.
- VHIT TESTING:
- VOR asymmetry using the HIMP of more than 20% on the horizontal semicircular canal (HSC).
- Affected side with HSC VOR gain ≥ 0.8.

The device used for the vHIT study was an ICS Otometrics by Natus^®^ (Godtrup, Denmark). Otosuite Vestibular software (version 4.1) was used to detect the VOR and CS/AS. A trained operator performed ten random right and left impulse movements in the horizontal plane on each patient’s head. These movements had a low amplitude (approximately 15°) and high velocity (150–200°/s). The patients were instructed to fixate on a wall target 1.5 m away during the HIMP and SHIMP stimulations (wall-fixed target in HIMP and head-moving target in SHIMP). Then, the AS patterns after the vHIT in SHIMP mode were examined on both the affected and unaffected sides.

**Figure 1:**
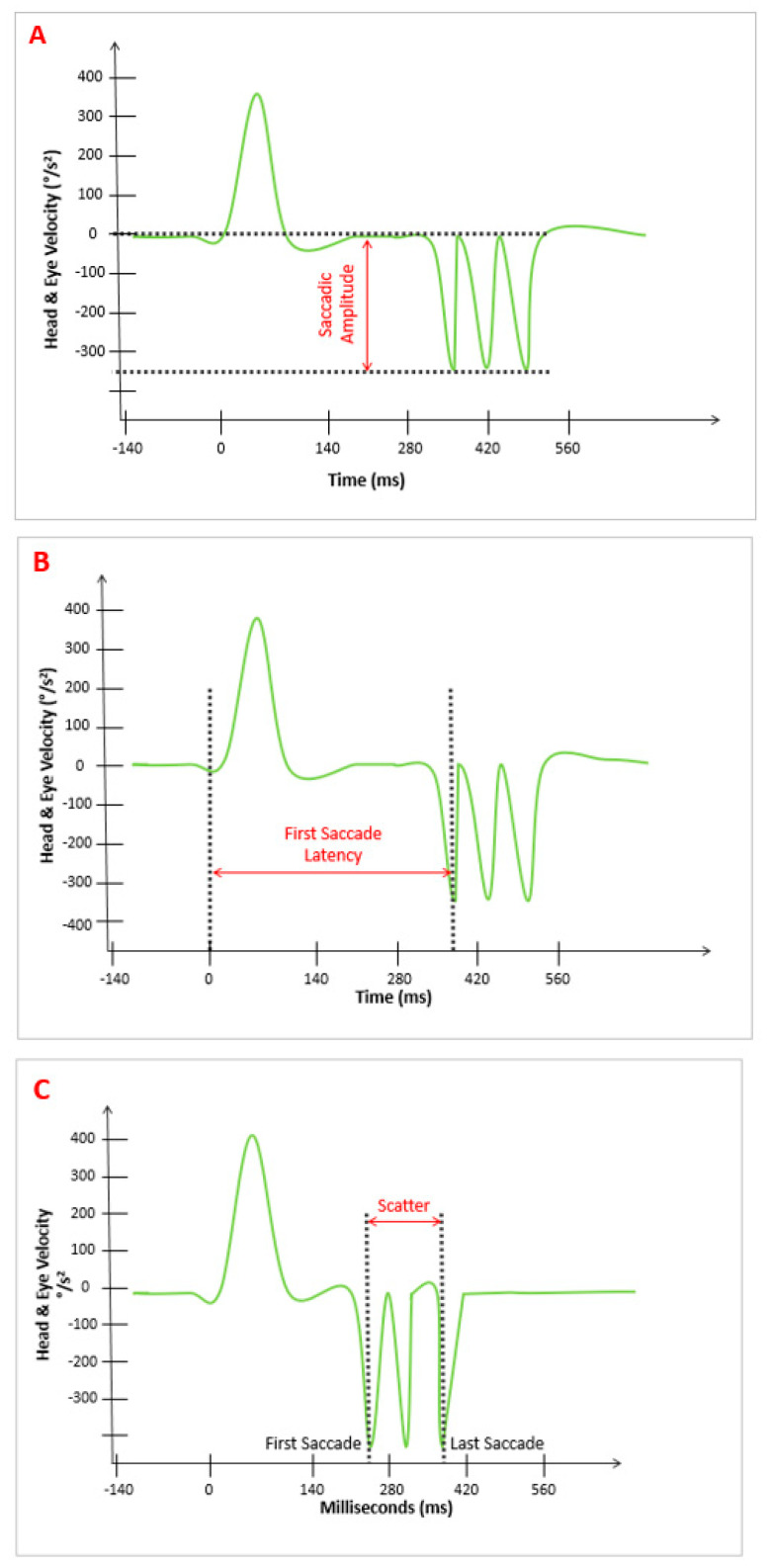
Different SHIMP AS patterns. (**A**) Shows that the parameter “Amplitude” is the value from the baseline to the widest AS saccade, expressed in °/s. (**B**) Shows the parameter “Latency,” which is the time from the start of the VOR complex (time 0) to the first AS, expressed in milliseconds (ms). (**C**) Shows the parameter “Scatter,” which is derived from the difference between the last and the first AS, expressed in milliseconds (ms). All the values were provided by the device. The green line represents the eys’s movement, generating VOR-complex and ASs.

We identified the following patterns in the ASs:

(1) “Amplitude pattern” (A): The amplitude of the AS that appeared after the SHIMP VOR complex. The device provided the average value of parameter “A,” which was expressed in °/s (Figure 1A).
(2) “Latency pattern” (L): The time at which the first AS appeared after the SHIMP VOR complex started. The average of the values of parameter “L” was provided by the device and was expressed in ms (Figure 1B).
(3) “Scattered pattern” (S): The time duration between the appearance of the first and last AS after the VOR complex on the stimulated side. The average value of parameter “S” was provided by the device and was expressed in ms (Figure 1C).

**Figure 2:**
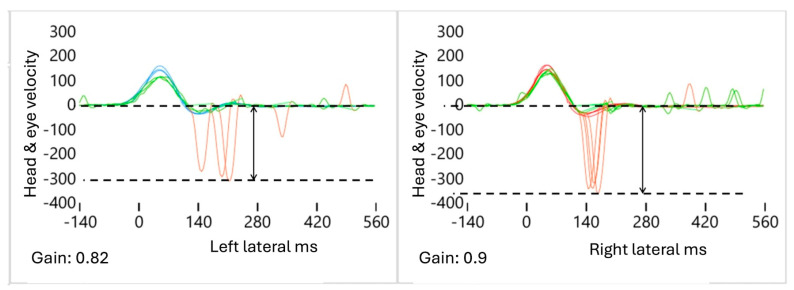
Decreased amplitude pattern A. In this example, the **left** side is considered pathological, with slightly reduced VOR and AS amplitude values compared to the **right** side, which is considered healthy. Blue: left head velocity (°/s); orange: right head velocity (°/s); green: left and right eye velocity (°/s); red: AS patterns (°/s and ms). The upper dotted line represents the baseline, and the bottom dotted line the highest value of AS Amplitude. The arrows represent the range between the baseline and the highest AS value.

**Figure 3:**
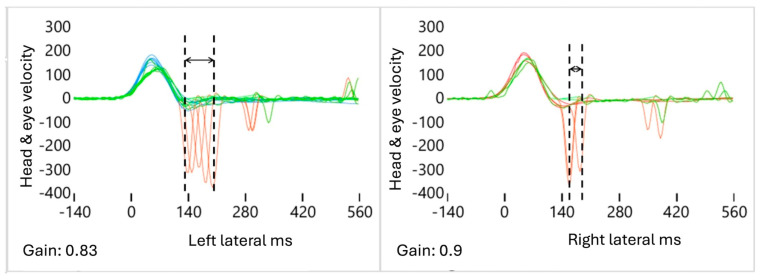
Increased scattered pattern “S”. In this example, the **left** side is considered pathological due to the increased interval between the first and the last AS compared to the **right** side, which is considered healthy. Blue: left head velocity (°/s); orange: right head velocity (°/s); green: left and right eye velocity (°/s); red: AS patterns (°/s and ms). The dotted lines represent the first and last AS. The arrows represent the interval between the first saccade (FS) and the last saccade (LS), defining the “Scattered pattern”.

**Figure 4:**
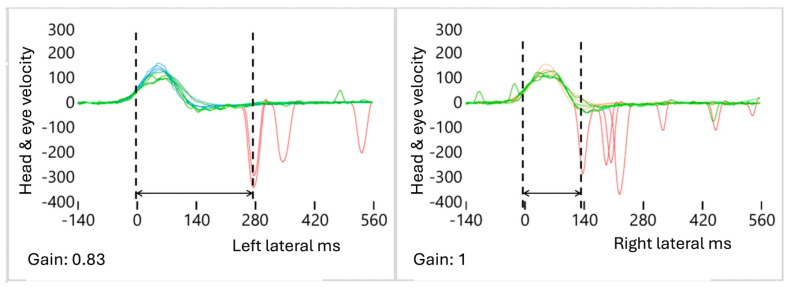
Increased Latency Pattern “L”. In this example, the **left** side is considered pathological due to the increased interval between the start of the VOR complex (time 0) and the first AS compared to the **right** side, which is considered healthy. Blue: left head velocity (°/s); orange: right head velocity (°/s); green: left and right eye velocity (°/s); red: AS patterns (°/s and ms). The first dotted line on each side represents the “time zero”at the start of the VOR-complex and the second dotted line represents the value of the first AS (ms). The arrows represent the latency time when the first AS appears compared to “time zero”.

A total of three alterations were identified in the AS patterns on the affected side (AFS) (Figure 2, Figure 3 and Figure 4).

(1) Decreased A: The ΔA value is defined as the difference in the average AS amplitude between the affected (AFS) and healthy (HTS) sides, calculated using the formula AAFS − AHTS = ΔA. As the VOR gain value decreased, we observed that the amplitude of the “A” values also decreased (Figure 2). In the case of mAUVL, slightly decreased VOR values produced slightly low “A” values that were present but not very sensitive.

(2) Increased S: The degree of the “S” pattern of the AS on both sides is obtained by calculating the difference in the AS latency between the first saccade (FS) and the last saccade (LS) of the AFS, compared to the contralateral HTS. This is calculated using the formula (FSAFS-LSAFS)-(FSHTS-LSHTS), which equals ΔSAFS-ΔSHTS, or “S”. As the VOR gain value decreased in the AFS, even slightly as in mAUVL, we observed that the “S” parameter values increased significantly ipsilaterally compared to the contralateral HTS (Figure 3).

(3) Increased L: The latency time between the start of the SHIMP VOR complex and the FS was significantly increased in the AFS compared to the contralateral HTS. It is calculated using the formula FSAFS − FSHTS = ΔFS (Figure 4).

In Group 1, the AFS corresponded to the side with the lower borderline HSC VOR-HIMP gain. In Group 3, the side with the lower HSC VOR-HIMP gain was compared with the side with the higher gain (HTS).

Then, a comparative analysis was conducted on the data from the three groups according to the same AS patterns (A, S, and L).

The analysis was performed using Jamovi software, version 2.7.2.0. A Student’s *t*-test was used to compare two groups on one continuous variable. When the continuous variable violated the normality assumption, the Mann–Whitney U test was used instead. The Shapiro–Wilk test was used to evaluate the normality assumption. A *p*-value of less than 0.05 was considered statistically significant. The values for the continuous variables are expressed as the mean and standard deviation and as the median and interquartile range (IQR).

The data collection was carried out in accordance with the Declaration of Helsinki guidelines, and the study was reviewed and approved by the Institutional Ethics Committee.

## 3. Results

A total of 104 patients with mild vertigo symptoms were observed during the specified period. Thirty patients were excluded from the study due to the application of exclusion criteria. The remaining 74 patients formed Group 1, consisting of 31 males and 43 females. The mean age of this group was 59 years (range: 13–85 years). The median VOR of the affected side was 0.81 (IQR 0.08); the median VOR of the healthy contralateral side was 0.93 (IQR 0.10). Group 2, which included 20 individuals aged 38–75 years, met the diagnostic criteria for sAUVL as outlined in the literature [2]. The median VOR of the affected side was 0.49 (IQR 0.17); the median VOR of the contralateral healthy side was 0.89 (IQR 0.21). Group 3, which comprised 20 HSs aged 22–63 years, exhibited no symptoms or signs indicative of active peripheral [2] or central [13] vestibular pathology. The median VOR of the side with the lower VOR was 0.89 (IQR 0.08), and the median VOR of the contralateral side was 0.96 (IQR 0.06). Most patients exhibited multiple saccadic patterns simultaneously.

The following results were obtained from the statistical analysis (Table 1 and Table 2):

Group 1: The A values of the ASs decreased by 13% on the affected side compared to the contralateral side. The S values increased by 123% on the affected side compared to the contralateral side. The L values increased by 30% on the affected side compared to the contralateral side.

Group 2: The A values of the ASs decreased by 30% on the affected side compared to the contralateral side. The S values increased by 84% on the affected side compared to the contralateral side. The L values increased by 27% on the affected side compared to the contralateral side.

Group 3: The A values of the ASs decreased by 7% on the side with the lower VOR. The S values increased by 14% on the side with the lower VOR. The L values increased by 10% on the side with the lower VOR.

**Figure 5:**
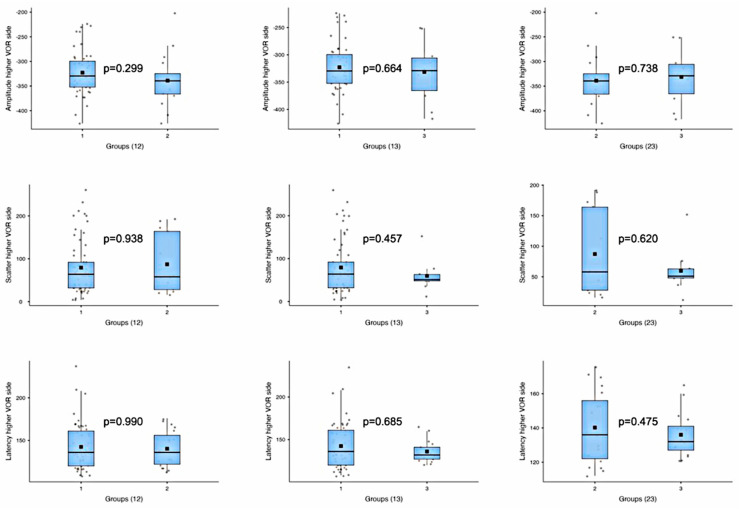
The boxplots show group-by-group comparisons of the amplitude, scatter, and latency parameters of anti-compensatory saccades in the ears with the higher VORs among the three groups.

**Figure 6:**
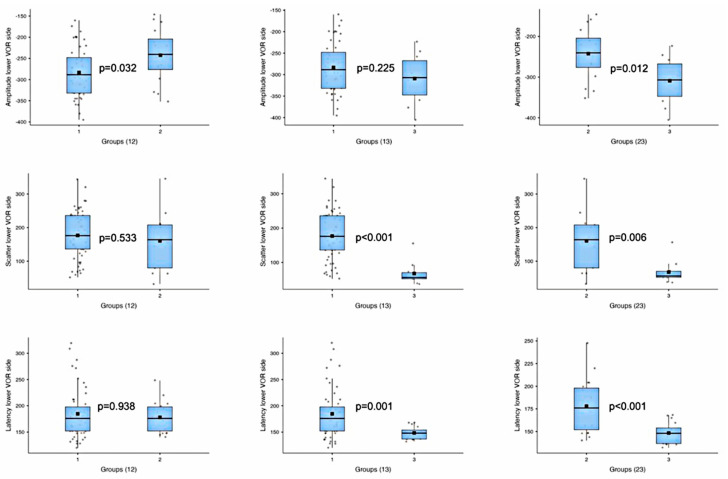
The boxplots show group-by-group comparisons of the amplitude, scatter, and latency parameters of anti-compensatory saccades in the ears with the lower VORs among the three groups.

The analysis of the A, S, and L parameters of the side with the higher VOR among the groups revealed no significant differences (Table 3, Figure 5). In contrast, a comparison of the sides with the lower VOR among the groups demonstrated statistically significant differences between Groups 1 and 2 for A, between Groups 1 and 3 for S and L, and between Groups 2 and 3 for all the parameters (Table 3, Figure 6).

## 4. Discussion

In AUVL, vHIT stimulation in the HIMP mode allows for the identification of two types of CS (i.e., corrective eye movements that compensate for VOR weakness): overt saccades, which are saccadic movements performed after head movement ends, and covert saccades, which occur before head movement ends [12,14,15,16]. Furthermore, depending on whether it is earlier or recent, the HIMP saccadic pattern can be “gathered” or “scattered”, respectively. Specifically, a “gathered” pattern indicates that the corrective saccades are tightly grouped and occur at a consistent time after the head impulse, which may suggest a less severe vestibular deficit. Conversely, a “scattered” pattern, in which the saccades are more dispersed and less predictable, may suggest more significant vestibular impairment or compensatory strategies [12].

Since its introduction, the SHIMP mode of vHIT has been an effective tool for assessing canal–ampullary dysfunction during high-frequency impulsive head stimulation (5–6 Hz) [8]. Unlike the classic HIMP, where the target is fixed (“earth-fixed”), the SHIMP involves a target (laser light) that moves with the head’s impulse. This allows for the generation and calculation of the VOR, which correlates directly with the HIMP VOR. However, the VOR gain values on SHIMP may be slightly lower compared with those on HIMP, due to the absence of saccades within the VOR complex and other probable central factors that are not yet fully elucidated [8]. An impulsive test according to the SHIMP modality induces large, easily identifiable ASs in healthy individuals. These saccades are oriented in the direction of stimulation and in the opposite direction of the VOR complex [6]. SHIMP and HIMP stimulation produce complementary saccadic patterns. In AUVL, CSs appear during HIMP stimulation and ASs progressively decrease during SHIMP stimulation, even disappearing completely in the most severe cases. The pathophysiological significance of CSs during HIMP stimulation and ASs during SHIMP stimulation differs greatly because SHIMP ASs are physiological, while the saccades during HIMP stimulation are pathological. This relationship has been exhaustively demonstrated in AUVL [6,9,10,11,12,14].

Therefore, identifying and analyzing the SHIMP AS patterns could be a reliable indicator of canal dysfunction. According to some authors, it could also guide the type, frequency, and intensity of rehabilitation exercises in AUVL patients [12]. In daily clinical practice, a wide variability in SHIMP AS presentation is frequently observed. Our goal was to diagnose AUVL by identifying the type and asymmetry of the AS patterns between the two sides, even when the VOR gain values are borderline.

The first parameter we considered was A, the average amplitude reduction in the ASs on the affected side compared to the unaffected side, in all subject groups. The vHIT device automatically calculated this value by averaging the saccadic peak rates, expressed in degree per second (°/s). In AUVL, the SHIMP AS amplitude decreases proportionally to the VOR gain loss, the intensity of subjective vertiginous symptoms, and the scattered pattern of CSs in the HIMP stimulation mode [11]. It has been well proven that the AS amplitude decreases with the VOR gain until it disappears completely in cases of labyrinthine areflexia [9,10,11]. Our results confirmed that the AS amplitude reduces proportionally to the VOR, indicating canal dysfunction.

A statistically significant reduction in the AS amplitude was observed in the ear with the lower VOR versus the contralateral ear in Groups 1 and 2 (Table 2). However, Group 3 did not show a significant reduction in the AS amplitude in the ear with the lower VOR (Table 2). Unfortunately, the difference in the reduction in the ear with the lower VOR between Group 1 and Group 2 was significant (Table 3, Figure 6), and the difference in the reduction in the ear with the lower VOR between Group 1 and Group 3 was not significant (Table 3, Figure 6). These findings indicate that variation in the AS amplitude is a useful, though insufficient, parameter for confirming the diagnosis of AUVL, since the VOR alterations in the VHIT-SHIMP stimulation modality and the consequent AS amplitude reduction are minimal. Conversely, a marked reduction in the VOR gain and AS amplitude is almost always evident in sAUVL (in over 80% of Group 3 patients in our study). This makes the parameter reliable for confirming canal dysfunction only when the deficit magnitude is evident (VOR gain < 0.6 and <0.4).

The second parameter we considered was S, which is the difference in latency (ms) between the first and last AS on the AFS relative to the contralateral side. Groups 1 and 2 showed a significant increase in the AS distribution compared to the normal side (Table 2). The median S value of Group 3 did not differ significantly between the ears with the higher and lower VORs (Table 2). The difference in the S values between the ears with the lower VORs in Groups 1 and 2 was not statistically significant (Table 3, Figure 6). The difference in the S values was statistically significant between Group 1 and Group 3 (Table 3, Figure 6), as well as between Groups 2 and 3 (Table 3, Figure 6). These results suggest that an increased S value could be a relevant, statistically significant parameter for diagnosing mAUVL.

The third parameter we considered was L, the latency difference from the beginning of the SHIMP VOR complex to the FS on each side. In this case, mAUVL and sAUVL showed significantly higher L values for the AFS than the contralateral HTS side: 30% for Group 1, 27% for Group 2, and 10% for Group 3. The difference in the L values between the ears with the lower VOR in Groups 1 and 2 was not statistically significant (Table 3, Figure 6). The difference in the L values was statistically significant between Groups 1 and 3, as well as between Groups 2 and 3 (Table 3, Figure 6). However, increased values of L alone are not useful for recognizing AUVL when there are borderline and/or similar VOR gain values between the two ears. This is because even in Group 3, the difference between the two ears was statistically significant (Table 2).

The VOR values and saccadic parameters (A, S, and L) are not directly interdependent and may or may not coexist. The reasons why some patients manifest saccadic changes in A, S, or L while others do not is unknown. Future studies and further multiparametric analyses of saccadic patterns and the clinical characteristics of patients from a more representative sample could provide insight into this phenomenon.

In our study, we also observed that the SHIMP-AS underwent modifications from a “gathered” to a “scattered” type for the side affected with AUVL (AFS) [12].

To confirm a definite VHIT diagnosis of mAUVL, we suggest performing the test in both the HIMP and SHIMP modalities and taking all the AS patterns into account.

Our study has some limitations. First, general and individual factors (e.g., age, CNS neuroplasticity, general vascular and endothelial structure, concomitant therapies, and iatrogenic and traumatic outcomes) could have interfered with the HIMP and SHIMP VHIT data and the pharmacological/rehabilitative management of patients. Other limitations, such as individual susceptibility to stimuli, patient lifestyle, visual dependence, and postural disturbances (including disorders secondary to an altered vestibulo-spinal reflex), may have interfered with the precise bedside and VHIT data collection [17]. Due to the small size of our sample, we could not perform a reliable subgroup analysis to investigate the influence of these confounding factors on the AS patterns, and the size discrepancies of the groups could have affected the statistical power of the study. We did not filter for these factors. Furthermore, we did not consider the time elapsed between symptom onset and the first vestibular assessment. A more precise selection of inclusion/exclusion criteria from a larger sample size is desirable.

All these considerations should be considered in future investigations to improve vHIT AS sensitivity and diagnostic specificity in doubtful clinical cases. We consider ours to be a preliminary study that should raise awareness among readers concerning the importance of SHIMP AS analysis in the daily clinical practice of otoneurologists to obtain an early and quick vHIT diagnosis of mAUVL, especially in borderline clinical cases. Certainly, following up with vHIT patients over time will provide important data on the subsequent therapeutic management (pharmacological and/or rehabilitative) and the evolution of vestibular compensation.

## 5. Conclusions

Analyzing AS patterns in SHIMP stimulation mode allows for greater diagnostic accuracy of vHIT for AUVL. The AS parameter “Amplitude” is a significant saccadic indicator in cases of severe AUVL. The “scattered pattern” parameter could be the main indicator of mild or borderline AUVL. The parameter “Latency time” can confirm canal dysfunction only when the scattered pattern is pathological.

Furthermore, given the diagnostic information that the suppression paradigm provides in cases of AUVL, a vHIT should be always performed in a combined HIMP+SHIMP mode, especially in cases of mAUVL.

## Figures and Tables

**Table 1:** Mean and median values of the anti-compensatory saccade patterns.

Variable		Group 1	Group 2	Group 3
Amplitude—lower VOR side	Mean ± Standard Deviation	−283.3 ± 57.5	−242.4 ± 63.4	−309.1 ± 58.2
	Median [IQR]	−288.5 [84.0]	−240.5 [72.0]	−307.0 [80.0]
Amplitude—higher VOR side	Mean ± Standard Deviation	−322.8 ± 45.3	−338.9 ± 54.7	−331.3 ± 56.5
	Median [IQR]	−329.5 [52.8]	−339.5 [41.5]	−329.0 [59.8]
Scatter—lower VOR side	Mean ± Standard Deviation	176.8 ± 69.0	160.5 ± 86.6	67.6 ± 34.9
	Median [IQR]	176.0 [100.0]	164.0 [128.0]	56.0 [18.0]
Scatter—higher VOR side	Mean ± Standard Deviation	79.3 ± 62.4	87.2 ± 68.5	59.8 ± 36.6
	Median [IQR]	64.0 [60.0]	58.0 [136.0]	51.0 [15.0]
Latency—lower VOR side	Mean ± Standard Deviation	184.7 ± 46.7	178.0 ± 29.1	148.4 ± 12.0
	Median [IQR]	176.0 [46.0]	176.0 [46.0]	148.0 [17.2]
Latency—higher VOR side	Mean ± Standard Deviation	142.4 ± 27.3	140.2 ± 21.0	136.0 ± 13.1
	Median [IQR]	136.0 [41.0]	136.0 [34.0]	132.0 [14.0]

Amplitude is expressed in degrees per second, while scatter and latency are expressed in milliseconds.

**Table 2:** Comparison of the anti-compensatory saccade patterns between sides in the same group.

Group	Tested Variables	N	Shapiro *p* (Lower)	Shapiro *p* (Higher)	Test	Statistic	*p*-Value
1	Amplitude—lower vs. higher VOR side	48	0.305	0.228	*t*-test	3.74	0.0003
2	Amplitude—lower vs. higher VOR side	16	0.663	0.421	*t*-test	4.61	<0.0001
3	Amplitude—lower vs. higher VOR side	10	0.820	0.560	*t*-test	0.87	0.398
1	Scatter—lower vs. higher VOR side	61	0.323	0.000012	Mann–Whitney U	3175.0	<0.0001
2	Scatter—lower vs. higher VOR side	13	0.640	0.018	Mann–Whitney U	131.0	0.018
3	Scatter—lower vs. higher VOR side	10	0.005	0.016	Mann–Whitney U	61.5	0.404
1	Latency—lower vs. higher VOR side	52	0.00029	0.00037	Mann–Whitney U	2189.5	<0.0001
2	Latency—lower vs. higher VOR side	19	0.235	0.165	*t*-test	4.59	<0.0001
3	Latency—lower vs. higher VOR side	16	0.142	0.152	*t*-test	2.78	0.009

N: number of subjects.

**Table 3:** Comparison of the anti-compensatory saccade patterns of the sides between groups.

Variable	Group	Test	Statistic	*p*-Value
Amplitude—lower VOR side	1 vs. 2	*t*-test	−2.28	0.032
	1 vs. 3	*t*-test	1.28	0.225
	2 vs. 3	*t*-test	2.74	0.012
Amplitude—higher VOR side	1 vs. 2	*t*-test	1.06	0.299
	1 vs. 3	*t*-test	0.45	0.664
	2 vs. 3	*t*-test	−0.34	0.738
Scatter—lower VOR side	1 vs. 2	*t*-test	0.64	0.533
	1 vs. 3	Mann–Whitney U	566.5	<0.001
	2 vs. 3	Mann–Whitney U	109.5	0.006
Scatter—higher VOR side	1 vs. 2	Mann–Whitney U	402.5	0.938
	1 vs. 3	Mann–Whitney U	350.5	0.457
	2 vs. 3	Mann–Whitney U	73.5	0.620
Latency—lower VOR side	1 vs. 2	Mann–Whitney U	500.5	0.938
	1 vs. 3	Mann–Whitney U	645.0	0.001
	2 vs. 3	*t*-test	4.05	<0.001
Latency—higher VOR side	1 vs. 2	Mann–Whitney U	492.5	0.990
	1 vs. 3	Mann–Whitney U	444.5	0.685
	2 vs. 3	*t*-test	0.72	0.475

## Data Availability

The data presented in this study are available on request from the corresponding author. The data are not publicly available due to privacy.

## Abbreviations

°/s	Degree Per Second
A	Amplitude Pattern
AFS	Affected Side
AS	Anti-Compensatory Saccades
AUVL	Acute Unilateral Vestibular Loss
BPPV	Benign Paroxysmal Positional Vertigo
CS	Compensatory Saccade
FS	First Saccade
HIMP	Head Impulse Paradigm
HIT	Head Impulse Test
HSs	Healthy Subjects
HSC	Horizontal Semicircular Canal
HTS	Healthy Side
IQR	Interquartile Range
L	Latency Pattern
LS	Last Saccade
mAUVL	Mild Acute Unilateral Vestibular Loss
ms	Milliseconds
S	Scattered Pattern
sAUVL	Severe Acute Unilateral Vestibular Loss
SHIMP	Suppression Head Impulse Paradigm
vHIT	Video Head Impulse Test
VOR	Vestibulo-Ocular Reflex
